# Differential Expression of Hepatic Genes of the Greater Horseshoe Bat (*Rhinolophus ferrumequinum*) between the Summer Active and Winter Torpid States

**DOI:** 10.1371/journal.pone.0145702

**Published:** 2015-12-23

**Authors:** Yanhong Xiao, Yonghua Wu, Keping Sun, Hui Wang, Bing Zhang, Shuhui Song, Zhenglin Du, Tinglei Jiang, Limin Shi, Lei Wang, Aiqing Lin, Xinke Yue, Chenji Li, Tingting Chen, Jiang Feng

**Affiliations:** 1 Jilin Provincial Key Laboratory of Animal Resource Conservation and Utilization, Northeast Normal University, Changchun, China; 2 Key Laboratory for Wetland Ecology and Vegetation Restoration of National Environmental Protection, Northeast Normal University, Changchun, China; 3 School of Life Science, Northeast Normal University, Changchun, China; 4 Core Genomic Facility, Beijing Institute of Genomics, Chinese Academy of Sciences, Beijing, China; 5 School of Life Science, Yunnan Normal University, Kunming, China; CSIRO, AUSTRALIA

## Abstract

Hibernation is one type of torpor, a hypometabolic state in heterothermic mammals, which can be used as an energy-conservation strategy in response to harsh environments, e.g. limited food resource. The liver, in particular, plays a crucial role in adaptive metabolic adjustment during hibernation. Studies on ground squirrels and bears reveal that many genes involved in metabolism are differentially expressed during hibernation. Especially, the genes involved in carbohydrate catabolism are down-regulated during hibernation, while genes responsible for lipid β-oxidation are up-regulated. However, there is little transcriptional evidence to suggest physiological changes to the liver during hibernation in the greater horseshoe bat, a representative heterothermic bat. In this study, we explored the transcriptional changes in the livers of active and torpid greater horseshoe bats using the Illumina HiSeq 2000 platform. A total of 1358 genes were identified as differentially expressed during torpor. In the functional analyses, differentially expressed genes were mainly involved in metabolic depression, shifts in the fuel utilization, immune function and response to stresses. Our findings provide a comprehensive evidence of differential gene expression in the livers of greater horseshoe bats during active and torpid states and highlight potential evidence for physiological adaptations that occur in the liver during hibernation.

## Introduction

Hibernation, in which organisms remain in a multiday torpor during winter, is an effective energy conservation strategy taken by endotherms to combat the adverse environment [1–3]. During torpor, the minimum body temperature of mammalian hibernators can drop below freezing and the minimum torpor metabolic rate can decrease to 4.3% of basal metabolic rate [4], which enables hibernators to conserve as much as 90% of their normal energy usage [5]. The liver, as a critical organ for metabolism, is likely to play a major role in physiological regulation during hibernation [6]. Energy metabolism during hibernation involves an important physiological transition in fuel utilization, i.e. shifting from carbohydrate oxidation to the catabolism of fat [7]. Lipid stored in white adipose tissue is hydrolyzed by lipase and converted to free fatty acids and glycerol during hibernation, a state of negative energy balance [6]. In the liver, glycerol and free fatty acids can be converted to glucose and ketone bodies, respectively. The ketone bodies can then be transmitted to other tissues as an energy source [8, 9].

The hibernation phenotype results from modulation of existing mammalian biochemical capabilities through the differential expression of existing genes [10], and much effort has been devoted to determining the genes that are differentially expressed during hibernation. In the liver, genes involved in carbohydrate, lipid and amino acid metabolism, detoxification and molecular transport were identified as differentially expressed between active and hibernating ground squirrels, with most of these genes down-regulated during hibernation [11]. In the American black bear (*Ursus americanus*), a large mammalian hibernator, comparison of microarrays between the livers of active and hibernating bears identified more than 300 differentially expressed genes; the majority of the genes that were over-expressed during hibernation play a role in protein biosynthesis and fatty acid catabolism while the genes with lower expression have roles in lipid biosynthesis and carbohydrate catabolism [12, 13]. Although these studies are valuable for increasing our understanding of the physiology of the liver in mammalian hibernators, animals used in these studies were limited to squirrels and bears, and little is known about the changes of gene expression in the liver of hibernating bats.

Bats (order Chiroptera) are the only mammals capable of sustained flight [14] and hibernating species exhibit a typical mammalian hibernation behavior. Because of bats’ small body size, high energy consumption when active, and limited fat storage capacity, winter hibernation is important for bats facing fluctuating food supplies (especially strictly insectivorous bats living in temperate regions) [1, 15]. Horseshoe bats from temperate regions are well-known hibernators [16, 17]. The greater horseshoe bat is a small insectivorous bat widely distributed in Europe, Africa, South Asia and Australia and China and has become a model species in the hibernation studies of bats [18, 19]. During hibernation their body temperature during hibernation drops from 40°C to 8°C and torpor bouts vary between 0.1 and 11.8 days, with individual means ranging from 1.3 to 7.4 days [1, 17]. Several studies have investigated the changes of gene expression in the brain of *R*. *ferrumequinum* in summer active and winter torpid episodes [20, 21], but there has been little effort to characterize changes in the transcriptome profile of the liver during hibernation, despite its critical role in a number of processes that are likely to be crucial for survival of hibernators. In general, there is a paucity of information on changes in the hepatic transcriptome during hibernation in bats: in their genomic study on the physiology and longevity of brandt’s bat (*Myotis brandtii*), Seim et al. (2013) present a short description of the gene expression in the liver of hibernating *M*.*brandtii* but detailed reports on the changes in hepatic gene expression in bats are still lacking [22].

In this study we sought to answer two main questions: 1) Are the changes in the liver transcriptome of hibernating *R*. *ferrumequinum* consistent with previous studies on other mammals? 2) Are the functions of the genes that are differentially expressed in the liver between active and torpid state similar to functions of the genes that are differentially expressed in the brain of *R*. *ferrumequinum*? To understand this, we sequenced the transcriptomes of liver tissues of active and hibernating greater horseshoe bats using the Illumina HiSeq 2000 platform to obtain a comprehensive of changes in hepatic genes expression of bats from active state to torpid state.

## Materials and Methods

### Ethics Statement

According to the regulations of Wildlife Conservation of the People’s Republic of China (Chairman Decree [2004] No.24), permits are required only for species included in the list of state-protected and region-protected wildlife species. *R*. *ferrumequinum* is not an endangered or region-protected animal species, so no specific permission was required. Sampling was conducted outside protected areas, with permission of the local forestry department. All experimental procedures carried out in this study were approved by the National Animal Research Authority of Northeast Normal University, China (approval number: NENU-20080416) and the Forestry Bureau of Jilin Province of China (approval number: [2006]178). All surgery was performed according to recommendations proposed by the European Commission (1997), and all efforts were made to minimize suffering of animals.

### Animals and Sample preparation

In this study, 16 female greater horseshoe bats were used. All individuals were caught in the Ground cave (125°50'25'' E, 41°4'8'' N) in Ji'an city, Jilin Province of Northeast China. Eight bats were captured in September 2011 (active state) and the others were captured in December 2011 (torpid state). The average weight of active bats was 23.44 ± 1.30 g, and that of torpid bats was 20.32 ± 2.18 g. It is quite difficult to get a time point during an inter-bout arousal, so only the summer active and winter torpid samples were collected in this study. Active individuals were transported to the laboratory and maintained under conditions of 21–22°C air temperature and 40% relative humidity, with food and water ad libitum. These animals were sacrificed after a total of 48 hours. Hibernating individuals were transported to the laboratory and placed in an artificial climate cabinet (HPG-280HX, HDL, China), with conditions of constant darkness, an ambient temperature of 5.5–5.7°C, 40–60% relative humidity and no food provided, and allowed to re-enter the torpid phase of hibernation. After 12 hours the bats were sacrificed, with a body temperature close to the ambient temperature (approximate 8.0°C) and having no response to stimulus (sound, touch and light).

All animals were euthanized by decapitation to minimize potential pain and suffering. Surgical procedures were promptly performed to protect RNA from degradation. Livers from active and torpid animals were rapidly excised, flash frozen in liquid nitrogen, and then stored at −80°C until processed for RNA isolation.

### cDNA library preparation and Illumina HiSeq 2000 procedure

Total RNA was isolated from liver tissues of bats at the active and torpid states at the same time using TRIzol Reagent (Life Technologies Inc., Carlsbad, CA) following the manufacturer’s protocol. Non-denaturing agarose gel electrophoresis and a NanoDrop spectrophotometer (Thermo Fischer Scientific Inc., Waltham, MA) were used to assess the quality and quantity of the isolated RNA, respectively. A260/280 values were all above 2.0, and electrophoresis of the RNA samples demonstrated that 28S and 18S rRNA were intact.

An Illumina TruSeq RNA library was constructed according to the manufacturer’s instructions, and 4 μg of total RNA were used to construct a cDNA library. Active and torpid libraries were tagged with different adapters and then sequenced on one lane using 2×100 nucleotide paired-end sequencing on the HiSeq 2000 platform. Raw sequence data generated were deposited into Short Read Achive (SRA) database of NCBI with the accession no. SRR2754983 (summer active state) and SRR2757329 (winter torpid state).

### Sequence assembly and RNA annotation

An in-house Perl script was used to remove sequencing adapters and PCR amplification reads. The first 20 bases of each paired-end read were compared and the best quality reads were reserved when the first 20 bases were identical. After processing the raw reads, trans-ABySS (v 1.3.2), a *de novo* short-read transcriptome assembly and analysis pipeline, was used to assemble reads [23]. In detail, first we ran multiple ABySS assemblies with k-mer range from 26 to 50 bp by the recommended parameters “abyss-pe E = 0 n = 5 v = -v k = $k OVERLAP_OPTIONS = '—no-scaffold' SIMPLEGRAPH_OPTIONS = '—no-scaffold' MERGEPATHS_OPTIONS = '—greedy'”. Trans-ABySS was then used to merge the different kmer assemblies into a single assembly. The choice of k-mer sizes depends on the read length of an RNAseq library. For reads lengths of 50 bp, we used 26 to 50 as suggested. To obtain a more reliable reference database for downstream analysis, processed reads of both active and torpid libraries were used to produce the assembly. In order to evaluate the transcriptome assembly, TransRate [24], a tool for reference-free quality assessment of *de novo* transcriptome assemblies, was used in our study. CD-HIT-EST, which is part of the CD-HIT package [25], was used to remove the shorter redundant transcripts, and the longest transcript was kept for each gene, with an empirical criteria of 95% similarity and 60% length coverage. Finally, the contigs were mapped to the genome of *M*. *lucifugus*, and contigs locating to the same site were merged into the longest sequence.

Gene information was obtained by BLAST searching gene sequences against the Nucleotide collection (nr/nt) database and the UniProt database with E-value < 10^−3^ cutoff.

### Raw reads mapping and quantification of expressed genes

Raw reads from active and torpid libraries were separately mapped to pre-assembled contigs (length > 500) using BWA v0.6.1-r104 [26, 27], with two critical parameters: less than five mismatches and no gap. Unique mapped reads were quantified into counts for each contig, which is considered to be a gene in this study. RPKM values (Reads Per Kilobase of transcript per Million mapped reads) were determined as the expression quantity of each gene [28].

### Differentially Expressed Gene (DEG) analysis and Quantitative Real-time PCR

In order to increase the reliability of the results, differential expression of genes in the liver of summer active and winter torpid *R*. *ferrumequinum* was analyzed using two methods, DEGseq [29] and GFOLD [30]. In the DEGseq method, the *P*-values calculated were corrected for multiple comparisons by using Benjamini-Hochberg method [31], which provides a *P*-value cutoff for significance which controlled by the false discovery rate (FDR) at 0.1%. In the GFOLD method, the *P*-value cutoff was fixed at 0.001. To create a list of differentially expressed genes of high confidence for our further analyses, a stringent criteria for differentially expressed genes, *P*-value<0.001(DEGseq), gfold-value≠0 and fold change >2, was used.

To test the validity of our measurements, qRT-PCR was performed to detect the relative mRNA expression level of 13 randomly selected genes which are down-regulated (*SDR42E1*, *TXK*, *ALAS2*, *NUP37*, *FGA*, *ARG1*, *CPB2*), up-regulated (*CCND2*, *DYRK1A*, *TAT*, *UCP2*), and not differentially expressed (*UFSP2*, *ZBED1*) during hibernation in the transcriptome sequencing. *β-actin* gene was selected as the house-keeping gene. The primer pairs for the 13 genes and house-keeping *β-actin* gene for *R*. *ferrumequinum* are listed in S1 Table, including their sequences, and product lengths. Messenger RNA samples from livers of 10 individuals (5 active, 5 torpid), which were randomly selected from the previous 16 individuals, were converted to cDNA templates. Quantitive real-time PCR was performed using StepOne Real-Time PCR System (Applied Biosystem) and an automatic threshold calculated by the StepOne software v2.1. For each sample, two technical replicates of each PCR reaction were run. For each target gene, reactions of all biological replicates (i.e., all samples) in the active and torpid state were completed on one plate to eliminate inter-run deviation. Each 10 μL PCR mixture reaction contained 5 μL THUNDERBIRD SYBR qPCR Mix (TOYOBO), 0.2 μL 50×ROX reference dye, 1 μL cDNA template and 0.25 μM of each primer. Then the PCR was performed under the following conditions: pre-denaturation at 95°C for 1min, then 40 cycles: 95°C, 15s; 60°C for 1min, with data collection after each cycle, followed by a melting curve. The amplification efficiencies of the house-keeping gene and 13 target genes were all between 90–100%. The standard deviation between two reactions of each sample was less than 0.5, so the mean *C*_T_ value of each sample was used in further analysis. The relative quantity was calculated by using 2^-ΔΔC^_T_ method [32] and the relative expression folds were expressed as mean ± S.E.M..

### Downstream functional analyses

Functional annotation was implemented by Gene Ontology (GO) and Kyoto Encyclopedia of Genes and Genomes (KEGG) pathway analyses using online wapRNA [33]. Downstream functional classification was achieved through integrated localization of GO [34] and KEGG pathway databases [35]. *P*-values were computed using the hyper-geometric test, and multiple test correction was performed using the Benjamini-Hochberg method [31] based on FDR cutoff of 0.05. In order to obtain more functional information about genes differentially expressed during torpor, differentially expressed genes used in functional analyses were defined using a less stringent criteria that *P*-value<0.001(DEGseq).

## Results

### Transcriptome sequencing, reads assembly and mapping

**Fig 1:**
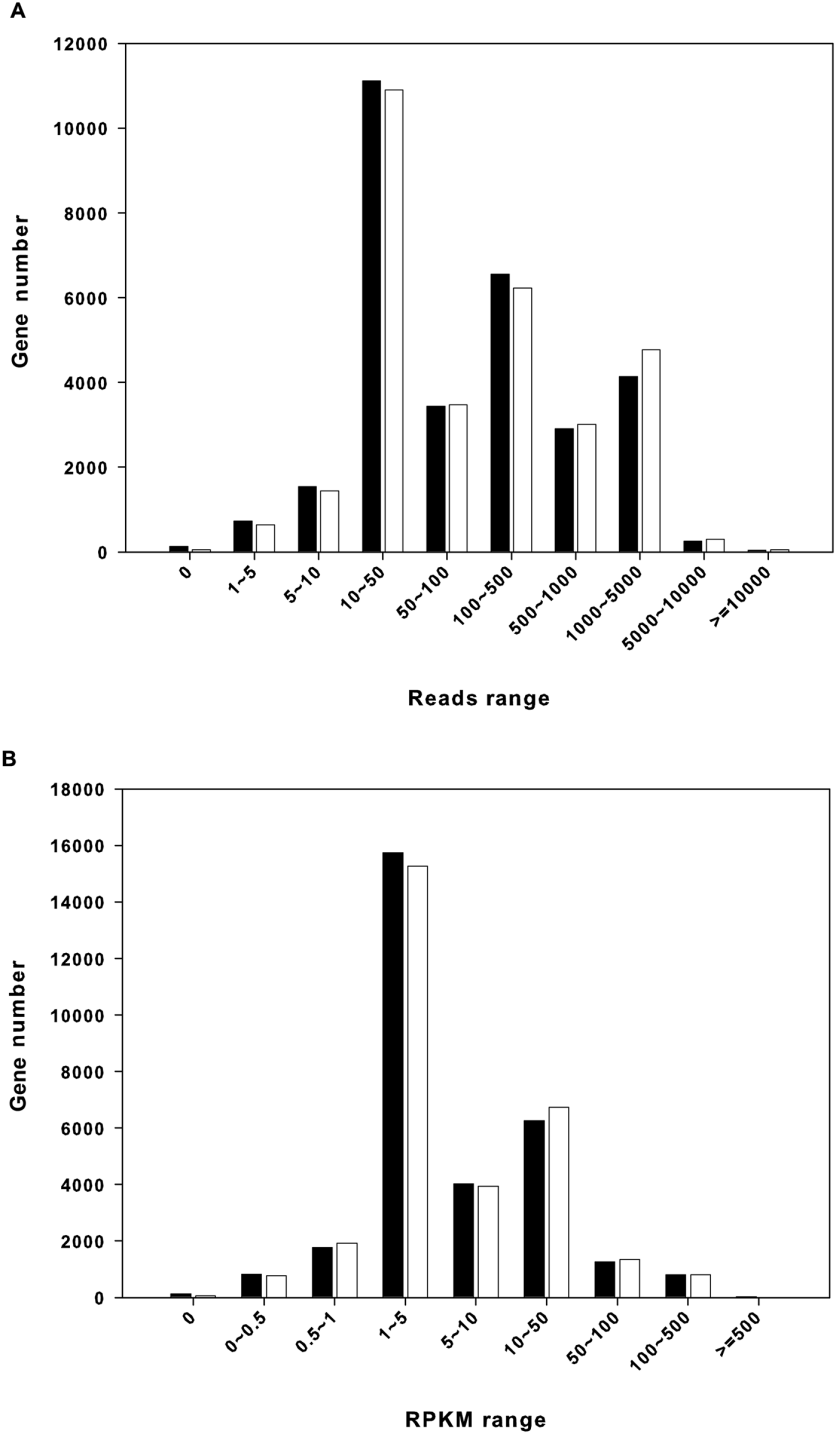
Interval distribution of gene expression abundance in the liver of *R*. *ferrumequinum*. (A) Gene expression abundance vs. Reads range. (B) Gene expression abundance vs. RPKM range. Solid bars show gene expression abundance in the active sample; open bars show gene expression abundance in the torpid sample.

Transcriptome sequencing produced 60,167,202 and 58,859,986 reads from active and torpid libraries, respectively, and the corresponding number of total bases generated was 6,076,887,402 and 5,944,858,586 bp. After *de novo* assembly using the Trans-ABySS program, we obtained 30,835 contigs (length >500 bp), with an N50 of 2,653 bp and an N90 of 735 bp (Table 1). The *TransRate* score of our *de novo* assembly was 0.18 (optimized score of 0.20). For further analyses of differential gene expression between active and torpid libraries, the raw reads of the two libraries were separately mapped to the assembled contigs (length >500 bp) that function as a transcriptome reference database. The results showed that 19,970,130 and 21,243,189 reads were mapped for active and torpid libraries, respectively, and the numbers of unique mapped reads were 16,301,491 and 17,718,379; the overall mapping rate was 41–47% (Table 1). Unique mapped reads were quantified into counts for each contig, and RPKM values, derived from the number of unique mapped reads were used to define the expression level of each gene. Fig 1 shows the interval distribution of gene expression abundance, which shows that genes having an RPKM value of 1–5 or 10–50 reads were the most abundant.

**Table 1:** Sequencing, assembly and mapping statistics of active sample and torpid sample.

	Active	Torpid
**Sequencing**		
Total Sequences (bp)	6,076,887,402	5,944,858,586
Sizes (Gb)	5.60	5.80
Total Reads	60,167,202	58,859,986
**Assembly**		
Contigs (>500bp)	30,835	
N50 (>500bp)	2,653	
N90 (>500bp)	740	
**Mapping**		
Total mapped reads (%)	41.35	46.96
Unique mapped reads (%)	33.75	39.16

The numbers of contigs (length >500bp), N50 and N90 were statistical results based on the sequence assembly of combined reads of active and torpid samples. Mapping statistics were results of raw reads mapping to contigs (length >500bp).

### Sequencing saturation and uniformity analysis

**Fig 2:**
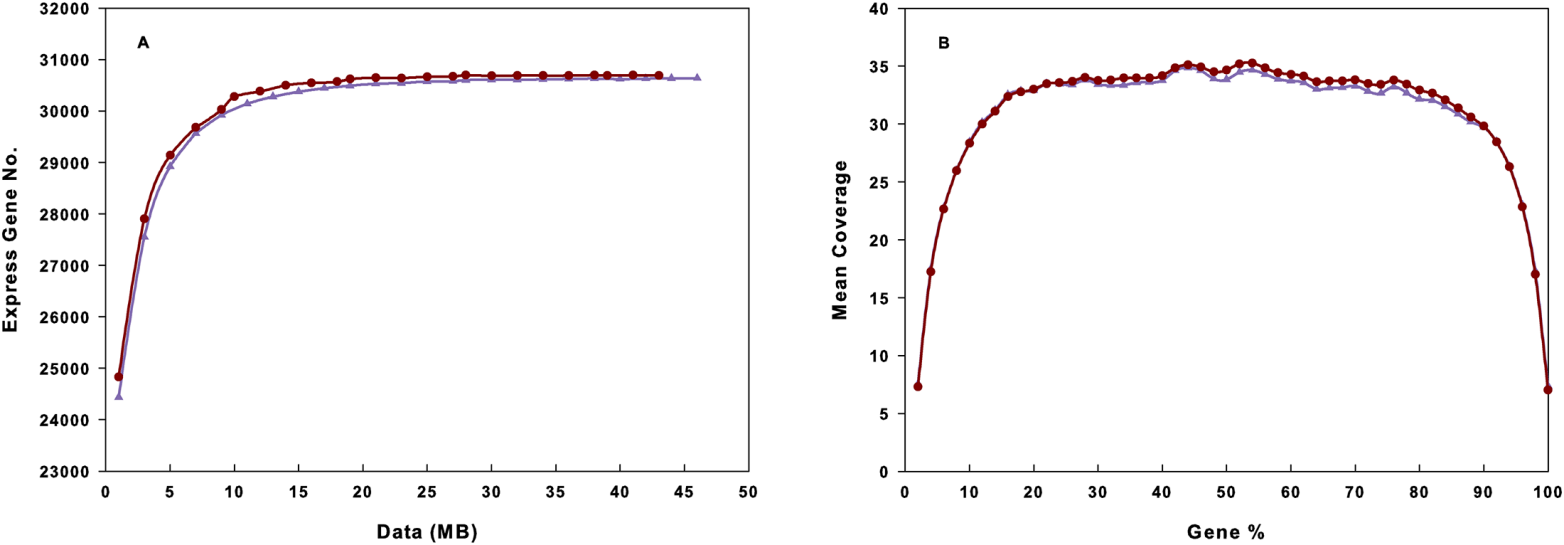
*R*. *ferrumequinum* liver transcriptome sequence saturation analysis and gene expression 5’~3’ bias analysis. (A) Sequence saturation analysis. (B) Sequencing 5’~3’ bias analysis. Dark red curve with solid circles represents active sample, and purple curve with solid triangles represents torpid sample.

To confirm whether the number of detected genes increased proportionally to the amount of sequence generated and to evaluate the quality of the libraries, saturation and 5’–3’ bias analyses were performed separately. Fig 2A shows a saturation trend where the number of detected genes almost ceased to increase when the number of reads reached 10 Mb and Fig 2B shows there was no 5’ or 3’ bias in the transcriptome sequencing and hence the data obtained had a good randomness.

### Identification and validation of differentially expressed genes (DEGs)

**Fig 3:**
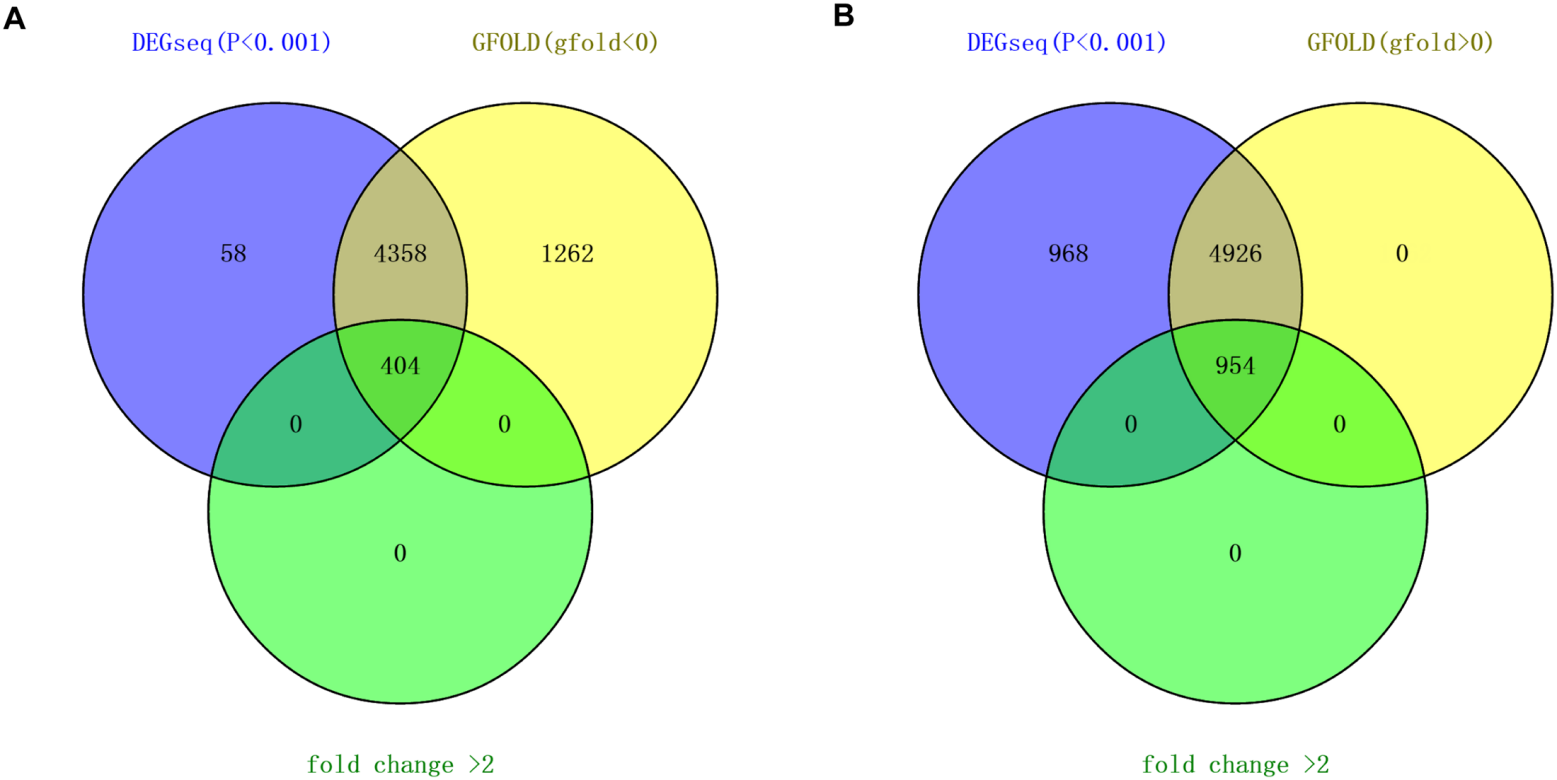
The number of differentially expressed genes obtained from DEGseq and GFOLD programs. (A) The number of down-regulated genes during torpor. (B) The number of up-regulated genes during torpor.

Applying a filter of *P*<0.001(DEGseq), gfold-value≠0 and fold change>2, 1358 significantly differentially expressed genes were identified between torpid and active liver samples, within which 404 genes are down-regulated and 954 genes are up-regulated in the torpid state (Fig 3, S2 Table). Considering genes highly expressed in the liver may have important roles in the physiological function of the liver, the genes with the top 10 RPKM values that are differentially expressed in the active and torpid states are listed in Table 2. Among genes that are significantly up-regulated in the torpid livers, the gene with the maximum RPKM value in the liver was *FABP1* encoding fatty acid binding protein 1, which can bind free fatty acids and is involved in intracellular lipid transport. The liver isoform of FABP in hibernators is adapted to function at low temperatures [36], indicating this enzyme is of importance in lipid metabolism during torpor. In addition, another up-regulated gene with high expression in the liver during torpor, *UCP2*, encoding uncoupling protein 2, is a member of the mitochondrial anion carrier proteins, functioning as a metabolic switch that enables the promotion of fatty acid metabolism over glucose utilization [37]. Conversely, among genes significantly down-regulated in the torpid state, the gene with the maximum RPKM value in the active liver sample was Cytochrome P450, family 1, subfamily A, polypeptide 2 (*CYP1A2*), encoding an important enzyme involved in an NADPH-dependent electron transport pathway and functioning in the bio-activation of carcinogenic aromatic and heterocyclic amines [38], and participating in the metabolism and subsequent elimination of potentially toxic xenobiotics and endogenous compounds [11]. The repression of this gene in the torpid state indicates that the liver’s role in breaking down endogenous waste products is depressed during torpor.

**Table 2:** Up-regulated genes with top 10 RPKM at the winter topid state and down-regulated genes with top 10 RPKM at the summer active state.

Gene Symbol	Description	RPKM (Active)	RPKM (Torpid)	Gfold value
**Up-regulated genes at the torpid state**				
FABP1	Fatty acid binding protein 1, liver	171.968	367.217	0.882
APOA2	apolipoprotein A-II	132.423	331.358	1.295
UCP2	Uncoupling protein 2 (mitochondrial, proton carrier)	84.865	208.792	1.100
S100A12	S100 calcium binding protein A12	18.725	184.007	2.863
CFD	Complement factor D (adipsin)	80.355	176.771	0.927
S100A4	S100 calcium binding protein A4	50.680	157.381	1.325
HMOX1	Heme oxygenase (decycling) 1	63.984	146.669	0.991
VCAM1	Vascular cell adhesion molecule 1	12.237	137.927	3.234
COCH	Coagulation factor C homolog, cochlin	0.205	136.467	7.773
CSF1R	Colony stimulating factor 1 receptor	50.359	134.269	1.252
**Down-regulated genes at the torpid state**				
CYP1A2	Cytochrome P450, family 1, subfamily A, polypeptide 2	457.785	81.029	-2.398
MBL2	Mannose-binding lectin (protein C) 2, soluble	283.950	120.069	-1.221
NIPSNAP3A	Nipsnap homolog 3A	178.902	75.387	-1.209
STEAP4	STEAP family member 4	165.308	69.277	-1.255
TYMP	Thymidine phosphorylase	136.184	61.267	-1.118
HLA-DMA	Major histocompatibility complex, class II, DM alpha	122.747	42.996	-1.404
TP53INP1	Tumor protein p53 inducible nuclear protein 1	99.027	45.840	-1.093
GJB2	gap junction protein, beta 2, 26kDa	93.6412	42.594	-1.085
DPYS	dihydropyrimidinase	93.5481	45.075	-0.989
C11orf54	chromosome 11 open reading frame 54	87.4898	37.480	-1.154

Genes with positive/negative gfold values were up/down regulated during torpor.

**Fig 4:**
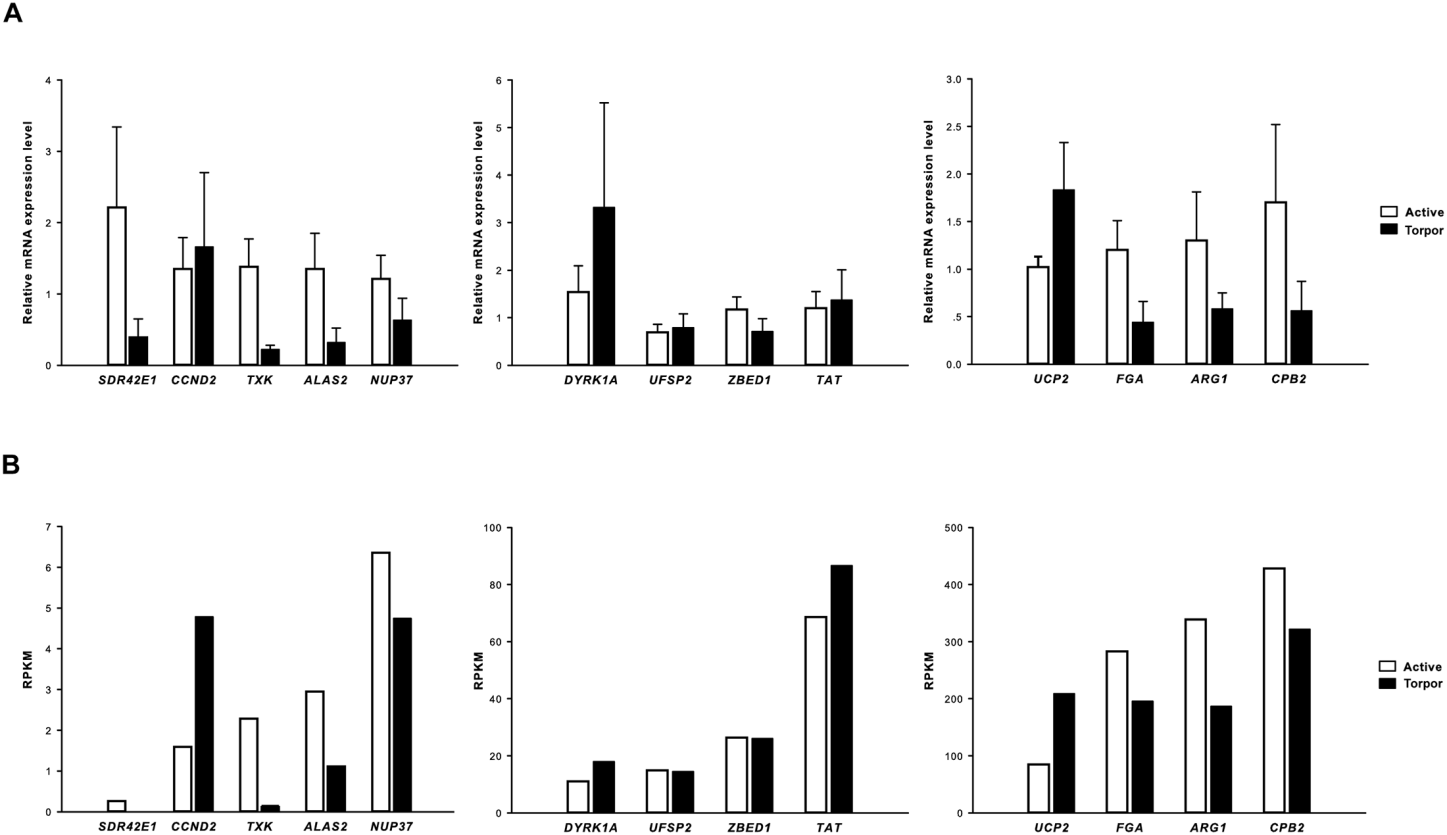
Comparison of 13 differentially expressed genes from RNASeq and qRT- PCR. (A) 13 genes expression from qRT-PCR. Results respresent mean + S.E.M. (N = 5) (B) 13 genes expression from RNASeq data. The correlation co-efficient between fold-changes of gene expression detected by qRT- PCR and RNASeq was 0.832 (*P*<0.01).

To test the validity of our measurements, we compared the RNASeq (RNA sequence) data of 13 randomly selected genes with the results of qRT-PCR experiments, which were used to detect the relative mRNA expression changes of the selected genes between active and torpid samples. Indeed, the highly significant correlation co-efficient of 0.832 indicated that the two independent measurements were consistent and show similar patterns, which ensured the reliability of the RNASeq data (Fig 4).

### GO and KEGG pathway enrichment analyses

**Fig 5:**
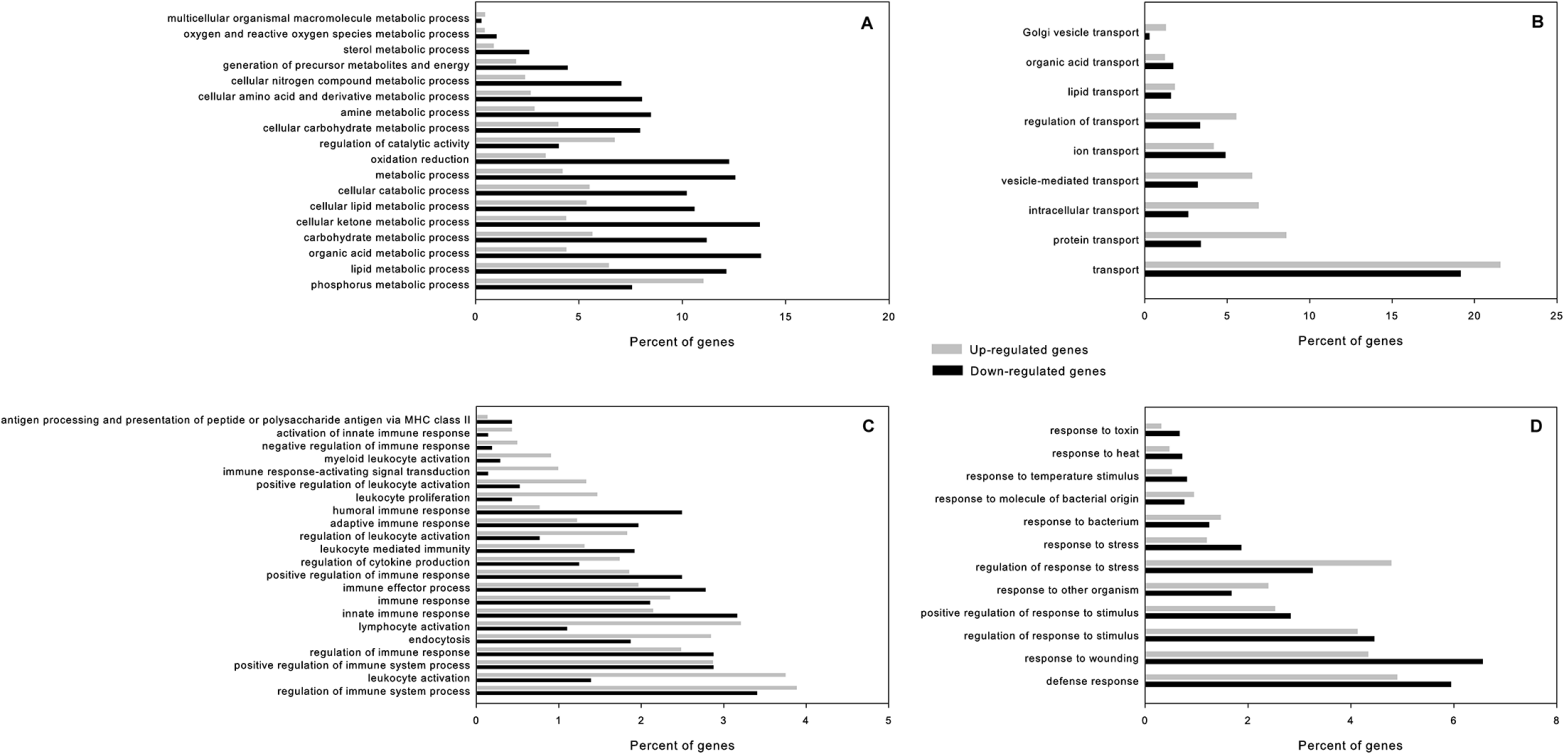
GO terms belonging to “biological process” annotated by most differentially expressed genes. (A) metabolic process (B) transport process (C) immune process (D) response process. Black bars show percent of genes down-regulated during torpor, Grey bars show percent of genes up-regulated during torpor.

To understand the functions of the differentially expressed genes, we carried out GO functional enrichment and KEGG pathway analyses. We identified 163 statistically significant GO terms, which were annotated by 2086 down-regulated genes and 4428 up-regulated genes in the torpid state respectively. The results were summarized into three main categories: biological process, molecular function and cellular component (S3 Table). In the category of biological process, GO terms, to which most of DEGs were annotated, can be classified into four categories: metabolic process, transport process, immune process, and response process (Fig 5). This result suggested these four biological processes play an important role in the hibernation of *R*. *ferrumequinum*.

**Fig 6:**
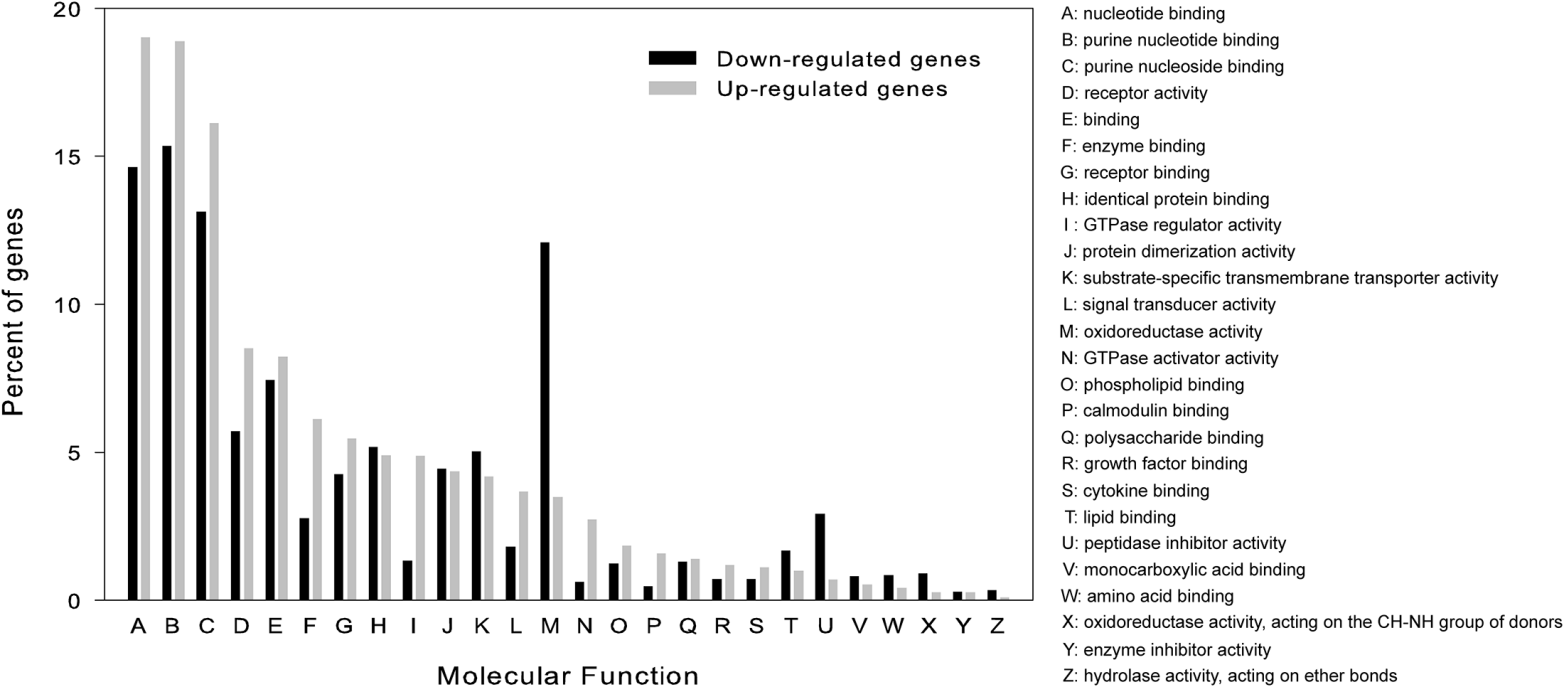
Histogram presentation of GO terms belonging to “molecular function” category. Black bars show percent of genes down-regulated during torpor, Grey bars show percent of genes up-regulated during torpor.

Among the 18 metabolic processes, the proportion of down-regulated genes during torpor in most metabolic processes was higher than that of up-regulated genes (Fig 5A), indicating a suppressed metabolism during torpor. In the transport processes, the proportion of up-regulated genes during torpor involved lipid transport and protein transport was higher than that of down-regulated genes (Fig 5B). Unlike metabolic processes, more immune processes had an overrepresentation of up-regulated genes than down-regulated genes in the torpid state (Fig 5C). Many differentially expressed genes were involved in response processes to wounding, stimulus, stress, and bacterium (Fig 5D). Percentages of up-regulated genes involved in “response to other organism”, “response to bacterium” and “response to molecule of bacterial origin” were greater than down-regulated genes during torpor. GO terms belonging to the category of molecular function are listed in Fig 6. Among 26 molecular functions, “nucleotide binding’, “purine nucleotide binding” and “purine nucleoside binding” were the three functions represented by the greatest number differentially expressed genes (Fig 6). In addition, “oxidoreductase activity” function was represented by a large percent of down-regulated genes in the torpid liver, indicating redox reaction may be depressed during torpor. We also found the molecular functions “signal transducer activity”, “calmodulin binding” and “cytokine binding”, were enriched in up-regulated genes in the torpid liver (Fig 6). The number of up-regulated genes involved in “lipid binding” was higher than that of down-regulated genes involved in this function though the percent of genes up-regulated during torpor was less than that of genes having lower expression during torpor (Fig 6 and S3 Table).

Besides presenting a comprehensive view of GO category enrichment, we have also listed the representative genes in some important GO categories in Table 3. These categories are associated with energy metabolism and response to stresses during torpor. In the carbohydrate metabolic process (GO: 0005975), genes involved in “glycolysis” such as *GCK*, *HK1*, *PFKFB3*, *PFKFB1*, *PYGM*, and *PFKP*, were significantly down-regulated in the torpid bats, while genes involved in “glycogen synthesis” and “gluconeogenesis” such as *GYS1* (glycogen synthase 1), *GYS2* (glycogen synthase 2), and *G6PC* (glucose-6-phosphatase), were up-regulated (Table 3 and S2 Table). Of these, hexokinase (*GCK* and *HK1*) and phosphofructokinase (*PFKP*, *PFKFB3*, and *PFKFB1*) are well-known rate-limited regulated enzymes of glycolysis, and glucose-6-phosphatase is a key enzyme of gluconeogenesis. The genes in the lipid metabolic process (GO: 0006629) that were down-regulated during torpor (*AGPAT2* and *ACSF3*) are involved in lipid biosynthesis while the up-regulated genes (*ACOT12*, *ACOX1*, *EHHADH*, and *SLC27A6*) are associated with fatty acid beta-oxidation, and fatty acid transport (Table 3 and S2 Table). Moreover, genes having “fatty acid binding” function in “lipid binding” (GO: 0008289) were all over-expressed during torpor (Table 3). In addition, lower-expressed genes in the torpid state involved in GO category of “cellular amino acid and derivative metabolic process” (GO: 0006519) have roles in protein synthesis (*PHGDH*, *ASS1*, *CBS*, and *PSAT1*), while over-expressed genes have roles in protein transport (*SLC7A6* and *APOA2*), and amino acid degradation (*PAH*, *GPT2*, and *HPD*). Finally, in the GO category of “response to stress” (GO: 0006950), we found many heat shock proteins (HSPs) and an oxidative stress responsive protein (*OXSR1*) were up-regulated during torpor. The HSPs including Hsp70 (HSPA4L, HSPA8), Hsp27 (HSPB7, HSPB1), Hsp90 (HSP90B1, HSP90AA1), Hsp105 (HSPH1), and Hsp40 (DNAJB4), function as molecular chaperones aiding in the assembly, folding, and translocation of various other proteins throughout the cells [39].

**Table 3:** Representative genes in several important Gene Ontology categories.

GO categories	Gene Symbol	Description	RPKM (Active)	RPKM (Torpid)	Gfold value
Carbohydrate metabolic process (GO: 0005975)					
	GCK	glucokinase (hexokinase 4)	4.819	0.702	-1.456
	HK1	hexokinase 1	22.893	6.899	-1.407
	PFKFB3	6-phosphofructo-2-kinase/fructose-2,6-biphosphatase 3	12.190	3.827	-1.241
	PFKFB1	6-phosphofructo-2-kinase/fructose-2,6-biphosphatase 1	20.800	7.201	-1.366
	PYGM	phosphorylase, glycogen, muscle	5.059	1.689	-0.751
	PFKP	phosphofructokinase, platelet	10.738	4.764	-0.734
	G6PC	glucose-6-phosphatase, catalytic subunit	14.265	38.304	1.370
	GYS2	glycogen synthase 2	2.708	6.473	0.937
	GYS1	glycogen synthase 1	1.230	3.697	0.250
Lipid metabolic process (GO: 0006629)					
	AGPAT2	1-acylglycerol-3-phosphate O-acyltransferase 2	11.313	3.715	-1.235
	ACSF3	acyl-CoA synthetase family member 3	5.412	1.670	-1.088
	SLC27A6	solute carrier family 27 (fatty acid transporter), member 6	0.310	5.511	2.228
	ACOT12	acyl-CoA thioesterase 12	5.082	11.520	0.683
	ACOX1	acyl-CoA oxidase 1, palmitoyl	1.004	2.556	0.659
	EHHADH	enoyl-CoA, hydratase/3-hydroxyacyl CoA dehydrogenase	1.121	2.605	0.327
Lipid binding (GO: 0008289)					
	FABP5	fatty acid binding protein 5	9.236	39.802	1.539
	FABP4	fatty acid binding protein 4, adipocyte	14.276	49.609	1.348
	FABP1	fatty acid binding protein 1, liver	171.968	367.217	0.882
	FABPH	Fatty acid-binding protein, heart	4.347	12.601	0.774
	FFAR2	free fatty acid receptor 2	0.548	2.964	0.553
Cellular amino acid and derivative metabolic process (GO: 0006519)					
	PHGDH	phosphoglycerate dehydrogenase	12.837	1.375	-2.447
	ASS1	argininosuccinate synthase 1	18.177	4.186	-1.843
	CBS	cystathionine-beta-synthase	8.222	2.704	-1.067
	PSAT1	phosphoserine aminotransferase 1	32.104	15.772	-0.727
	SLC7A6	solute carrier family 7 (amino acid transporter light chain, y+L system), member 6	2.927	9.865	1.308
	APOA2	apolipoprotein A-II	132.423	331.358	1.295
	PAH	phenylalanine hydroxylase	2.677	6.407	0.717
	GPT2	glutamic pyruvate transaminase 2	3.448	7.536	0.698
	HPD	4-hydroxyphenylpyruvate dioxygenase	2.591	5.434	0.385
Response to stress (GO: 0006950)					
	HSPH1	heat shock 105kDa/110kDa protein 1	0.108	3.053	2.779
	DNAJB4	DnaJ (Hsp40) homolog, subfamily B, member 4	0.139	1.816	1.582
	HSP90AA1	heat shock protein 90kDa alpha (cytosolic), class A member 1	14.705	38.995	1.145
	HSPA4L	heat shock 70kDa protein 4-like	5.917	12.814	0.978
	HSPA8	heat shock 70kDa protein 8	30.299	66.233	0.917
	HSPB7	heat shock 27kDa protein family, member 7	0.520	3.922	0.751
	HSPB1	heat shock 27kDa protein 1	1.301	4.598	0.649
	HSP90B1	heat shock protein 90kDa beta (Grp94), member 1	3.370	7.077	0.611
	OXSR1	oxidative-stress responsive 1	0.739	2.089	0.098

Genes with positive/negative gfold values were up/down regulated during torpor.

**Fig 7:**
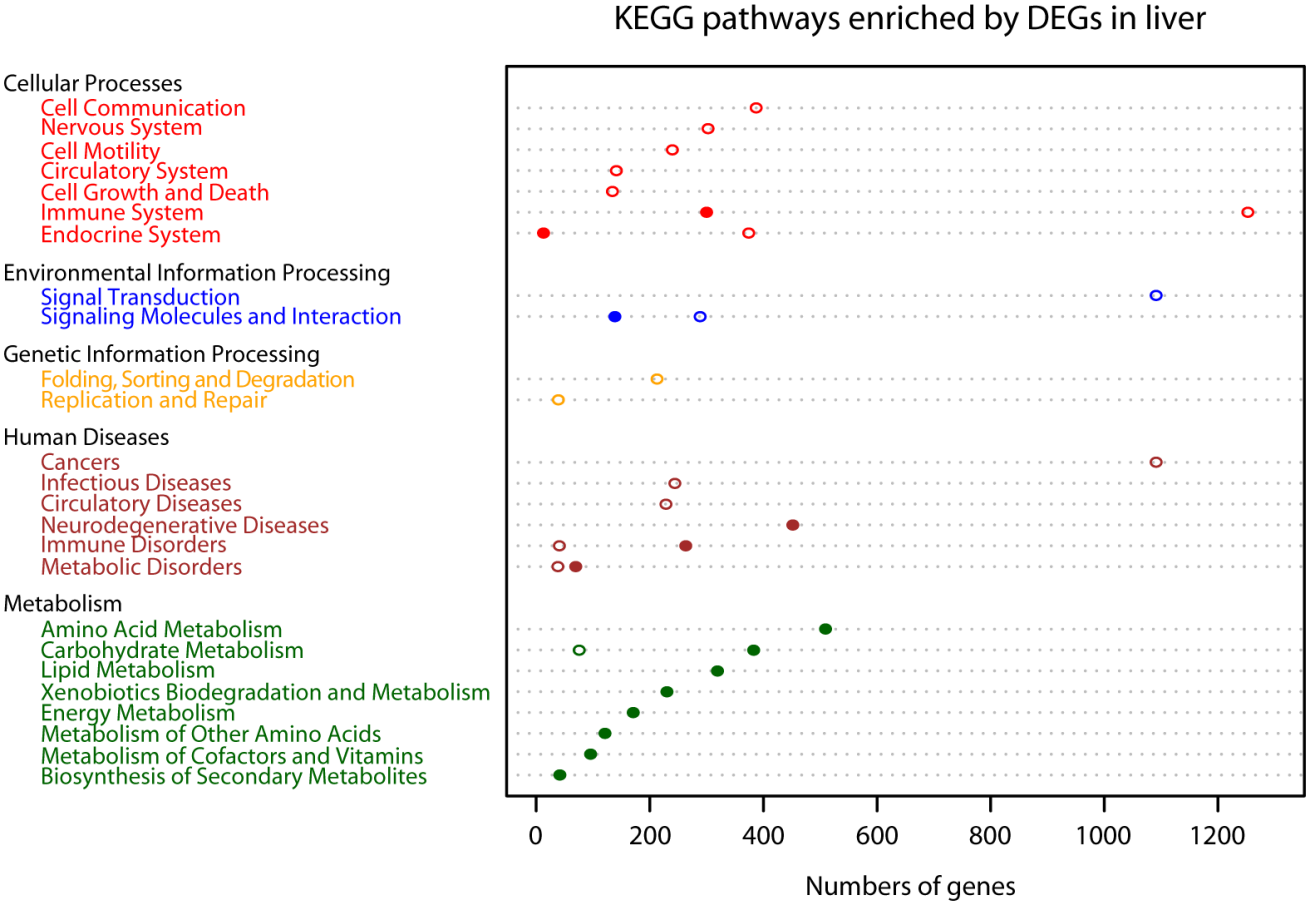
KEGG pathway enrichment analysis of differentially expressed genes. Pathways belonging to different classifications, including “Cellular Processes”, “Environmental Information Processing”, “Genetic Information Processing”, “Human Diseases” and “Metabolism”, were listed on the left of the plot. The number of genes enriched into each pathway was dotted on the corresponding dashed line. Filled circles show number of genes down-regulated during torpor, and hollow circles show number of genes up-regulated during torpor.

Through the KEGG pathway analysis, 8,377 DEGs were annotated to 242 pathways, but only 105 of these were significantly annotated (Benjamini-Hochberg adjusted *P*-value <0.05). These were classified into five categories: Cellular Process, Environmental Information Processing, Genetic Information Processing, Human Diseases, and Metabolism (S4 Table). Of these 105 pathways, 54 were significantly annotated by 1,226 down-regulated genes and 51 were significantly annotated by 3,288 up-regulated genes (S4 Table) in the torpid bats. To have a visual understanding of the results of the KEGG pathway enrichment analysis, we plotted the total number of DEGs enriched in each second class pathway by using R 3.0.3, as shown in Fig 7.

KEGG pathway analysis showed that 1,871 down-regulated genes during torpor were significantly annotated in 40 metabolism pathways, including amino acid metabolism, biosynthesis of secondary metabolites, carbohydrate metabolism, energy metabolism, lipid metabolism, metabolism of cofactors and vitamins, and xenobiotic biodegradation and metabolism, amounting to 60% of down-regulated genes (Fig 7 and S4 Table) in the torpid bats. Only inositol phosphate metabolism was significantly annotated by 76 up-regulated genes, amounting to only approximately 1% of up-regulated genes in the torpid bats (Fig 7 and S4 Table). Moreover, the KEGG pathway enrichment analysis showed that among all pathways significantly annotated by up-regulated genes during torpor, the immune system had the greatest number of significantly enriched genes (1,253). Ten immune-related pathways were identified, amounting to 20% of pathways significantly annotated by up-regulated genes during torpor (Fig 7 and S4 Table). The primary functions of these immune-related pathways included monitoring and obliterating invading antigens, such as bacteria, viruses and fungi, and the inflammatory response. In every immune-related pathway, most of the genes promoting immune activity were significantly up-regulated in the torpid state. However, there were two immune-related pathways that were significantly annotated by down-regulated genes in the torpid liver, including complement and coagulation cascades (ko04610) and antigen processing and presentation (ko04612); however the number of down-regulated genes enriched in the two pathways was obviously lower than that of up-regulated genes in the torpid liver enriched in immune-related pathways (S4 Table). In addition, three KEGG pathways, proteasome (ko03050), ubiquitin mediated proteolysis (ko04120) and DNA replication (ko03030) were found only enriched by up-regulated genes in the torpid state, which indicates an increase in the processes of ubiquitin-mediated proteolysis and DNA replication during torpor (Fig 7 and S4 Table). Moreover, enchanced cell activities during torpor were inferred from the increased expression of cell growth and death related genes regulating cell apoptosis (ko04210) and cell motility related genes regulating actin cytoskeleton (ko04810) (S4 Table). Three signal transduction pathways, MAPK (ko04010), ErbB (ko04012) and Notch signaling pathways (ko04330) were also found significantly enriched by up-regulated genes in the torpid state (S4 Table).

## Discussion

### Metabolic changes during torpor

As an important strategy for energy conservation, metabolic rate depression plays a critical role in many mammalian hibernators surviving harsh winter environments [36]. The minimum torpor metabolic rate during torpor can be decreased to 4.3% of basal metabolic rate [4], which enables hibernators to save as much as 90% of normal energy usage [5]. How can the hibernators adjust their metabolic rate from a normal level to such a low level? In biology, altered patterns of gene expression allow a diversity of phenotypes even with a common genotype [7]. The physiological changes that occur as an organism shifts between active and torpid states are likely the result of altered gene expression of proteins that serve specific functions. In this study, we found that most of the genes that were down-regulated during torpor were involved in metabolic pathways and the proportion of down-regulated genes in most metabolic processes were greater than that of up-regulated genes during torpor. Moreover, a greater proportion of genes involved in “oxidoreductase activity” were down-regulated in torpid bats than up-regulated. For example, *CYP1A2*, which encodes an important enzyme involved in an NADPH-dependent electron transport pathway that plays a crucial role in energy metabolism, was down-regulated during torpor. All of these results are consistent with metabolic depression during torpor and may indicate that transcriptional changes lead to metabolic adjustment between states. Supporting our findings, Lei et al. (2014) found co-downregulation of genes involved in glycolytic pathway play a central role in metabolic suppression during torpor in the brain of torpid *R*. *ferrumequinum*, which also suggests a systemic of suppression of metabolism during torpor.

Although metabolic processes are depressed overall, bats, like other mammals, still undergo critical shifts in their fuel utilization during torpor, specifically switching from carbohydrate- to fat-based metabolism. Hampton et al. (2011) found that the relative abundance of lipogenesis-related genes was higher in the August and October, while the relative abundance of lipolysis-related genes was higher in the hibernation seasons in the white adipose of the thirteen-lined ground squirrel (*Ictidomys tridecemlineatus*), which indicates lipids are the primary energy source during hibernation [40]. In general, torpid animals rely on fatty acids as their main energy source [41]. In our study, a significant reduction in the weight of torpid bats compared with active ones, with the loss of white adipose during hibernation (observed in the sampling process), confirmed the earlier findings.

Our finding that genes involved in lipid metabolism are differentially expressed in torpid animals is also consistent with findings from other mammals. In an early study on the golden-mantled ground squirrel (*Spermophilus lateralis*), the genes involved in lipid metabolism had a significant overrepresentation in the genes that were elevated during hibernation [11]. Similarly, studies on gene expression in the livers of bears indicated that genes involved in glycolysis, amino acid catabolism, *de novo* lipogenesis, the urea cycle and detoxification are down-regulated while gluconeogenesis-, β-oxidation- and ketogenesis-related genes that are involved in lipid metabolism and carbohydrate synthesis are up-regulated during hibernation [6]. Seim et al. (2013) also found many differentially expressed genes associated with the shift from carbohydrate to lipid metabolism between the livers of active and torpid *M*.*brandtii*.

We also found that genes encoding several key rate-limited regulated enzymes of glycolysis (*GCK*, *HK1*, *PFKFB3*, *PFKFB1*, and *PFKP*) were expressed at lower levels during torpor, and another gene encoding a key enzyme of gluconeogenesis (*G6PC*) was over-expressed in the torpid bats, suggesting lowered utilization of glucose during torpor. In addition, *PDK4* (pyruvate dehydrogenase kinase isoenzyme 4) was up regulated during torpor with a fold change of 1.56 (<2). Elevated expression of *PDK4* during torpor was also found in the heart, skeletal muscle, and white adipose of the thirteen-lined ground squirrel [40]. Its increased expression can inhibit carbohydrate metabolism by preventing the flow of glycolytic products into the tricarboxylic acid cycle [42, 43]. In our study, more genes involved in lipid and protein transport were over-expressed during torpor, and more genes involved in lipid binding were up regulated than down regulated. Those genes involved in lipid metabolism that were down-regulated in torpid animals were involved in lipid biosynthesis, while up-regulated genes in this GO category were associated with fatty acid beta-oxidation, and fatty acid transport. Moreover, genes having roles in fatty acid binding were also up-regulated during torpor. For example, *FABP1* had its maximum expression in the torpid liver. Similarly, the expression of liver fatty acid-binding protein (*FABPL*) was elevated 1.9-fold in the torpid liver of *S*. *lateralis* [11]. The liver isoform of FABP in hibernators is adapted to function at low temperatures [36], which can contribute to lipid transport during torpor. These results indicate a shift of primary energy source from carbohydrate to lipid. Moreover, another gene directly involved in lipid β-oxidation and ketogenesis, uncoupling protein 2 (*UCP2*), was up-regulated in the torpid library. This gene functions as a metabolic switch that enables the promotion of fatty acid metabolism over glucose utilization, and was over-expressed in several tissues of other mammals during hibernation, fasting and exposure to cold [5, 44, 45]. Over-expression of *UCP2* in a state of prolonged fasting could increase the concentration of fatty acids in the mitochondrial matrix, thereby preventing fatty acid accumulation and protecting against oxidative stress caused by reactive oxygen species [37]. Over-expression of *UCP2* also suggested that fatty acid catabolism is the primary energy source during torpor. Another two genes with fold changes <2, *CPT1A* (fold change = 1.48) and *HMGCS2* (fold change = 1.86), also have over-expression during torpor. CPT1A (hepatic carnitine palmitoyltransferase 1A), which catalyzes the transfer of the acyl group of long-chain fatty acid-CoA conjugates onto carnitine, an essential step for the mitochondrial uptake of long-chain fatty acids and their subsequent beta-oxidation, plays an important role in lipid metabolism [46, 47]. Expression of *CPT1A* was also increased during hibernation in ground squirrels [45]. HMGCS2 (mitochondrial 3-hydroxy-3-methylglutary-CoA synthase 2) is an important controller in the ketogenic pathway and functions as a rate-limiting enzyme [48]. Over-expression of *HMGCS2* and increased blood concentration of ketone bodies were found in hibernating bears [6].

### Unexpected changes in immune function during hibernation

In our study, many of the differentially expressed genes in the liver between active and torpid *R*. *ferrumequinum* were associated with immune function. Our GO enrichment analysis revealed that immune related genes were more likely to be up regulated than down regulated during torpor, especially genes related to cytokine binding. Similarly, the KEGG pathway analysis showed that the immune system had the greatest representation in terms of number of genes among pathways significantly associated with genes that are up-regulated during torpor. Further, 10 immune-related pathways were identified, amounting to 20% of pathways significantly annotated by up-regulated genes. Although two immune-related pathways (ko04610 and ko04612) were marked by a significant enrichment of down-regulated genes in the torpid state, the total number of down-regulated genes enriched in these two pathways was obviously lower than that of up-regulated genes enriched in immune-related pathways. At first it may be surprising that so many immune-related genes are differentially expressed in the livers of active and torpid *R*. *ferrumequinum*. However, the liver not only plays an indispensable role in metabolism, but also acts as an important immunological organ [49]. Eighty percent of blood supplied to the liver is from the gut, which is rich in bacterial products, environmental toxins and food antigens [50]. The liver plays a key role in innate immunity and acts as an organ barrier or a filter between the digestive tract and the rest of the body [50].

Our findings seemingly contrast with other studies of hibernation which found that hibernation depressed both the innate and adaptive immune systems, e.g., lower complement levels, reduced phagocytotic capacity, decreased cytokine production, and diminished lymphocyte proliferation and antibody production were observed [51]. Golden-mantled ground squirrels (*Callospermophilus lateralis*) in the torpid state did not present a febrile response when injected with lipopolysaccharide, while aroused animals did [52]. In addition, torpid thirteen-lined ground squirrels rejected a skin allograft three to four times slower than summer active animals [53]. However, little is known about the transcriptional expression of genes involved in immune functions of hibernating mammals. Given the fact that our results are inconsistent with previous descriptions of the effect of hibernation on the mammalian immune system, we speculated that immune activity may be organ-specific during hibernation. For example, the numbers of intraepithelial lymphocytes and lamina propria leukocytes were increased in the intestine of thirteen-lined ground squirrels during hibernation, and mucosal levels of IFN-γ, TNF-α, IL10 and IL4 were greater in torpid hibernators than in active animals, which may contribute to the preservation of epithelial integrity throughout the winter fast [54]. However, another organ, the thymus, which is vital to the immune system, was surrounded by brown adipose tissue and contains almost no lymphocytes during hibernation [55–57]. The enhanced immune activity that we have inferred in the liver may help to resist pathogen invasion. In GO analysis, we found that genes involved in “response to other organism’, “response to bacterium” and “response to molecule of bacterial origin” were up-regulated in torpid animals, indicating an enhanced defense response. Our KEGG pathway enrichment analysis revealed that a number of up-regulated genes in the torpid state were significantly enriched in “human diseases” pathways and three “infectious diseases” pathways including epithelial cell signaling in *Vibrio cholera* infection (ko05110), *Helicobacter pylori* infection (ko05120) and pathogenic *Escherichia coli* infection (ko05130) (S4 Table), suggesting bats are at high risk of pathogens infection during hibernation. A particularly pressing example of the vulnerability of hibernating bats to disease is that of white nose syndrome (WNS), a fungal disease that has decimated bat populations in North America [58] and is continuing to spread. Moore et al. (2013) found that little brown bats (*Myotis lucifugus*) hibernating in WNS-affected sites showed immunological changes, specifically elevated circulating leukocytes, which may be evident of attempted defense against *Geomyces destructans* [59].

### Molecular anti-stress response

During deep torpor, hypothermia and oxidative stress from ischemia-reperfusion may be highly stressful and may compromise the survival of the hibernator [7]. A long period of low body temperature has harmful effects on such processes as protein stability, membrane function, ATP synthesis, activity of key regulatory enzymes, and cytoskeletal integrity [60, 61]. Blood flow in the splanchnic organs is preferentially decreased during deep torpor. Upon arousal to euthermic body temperature, reperfused oxygenated blood induced by the increased metabolic activity in all cells will be strikingly stressful for splanchnic organs that are typically hypo-perfused [7]. This is because ischemia-induced oxidative stress during hibernation plays a crucial role in contributing to cell death [62]. The successful survival from these stresses suggests the existence of anti-stress responses. Genes involving “unfolded protein binding” and “protein folding” were over-expressed during torpor, and many heat shock proteins were differentially expressed in the brain of *R*. *ferrumequinum*, which suggests an adaptive response of torpid bats to environmental stress [21]. In this study, we found many up-regulated genes in the torpid liver library involved in response processes to stress and stimulus (e.g. heat, temperature), and the proportion of up-regulated genes involved in “regulation of response to stress” was greater than down-regulated genes. Moreover, genes encoding heat shock proteins (Hsp70, Hsp27, Hsp90, Hsp105, Hsp40) that were identified in the brain of torpid *R*. *ferrumequinum* were also over-expressed in the liver during torpor. Their induction during stress is believed to be important for preventing misfolding and aggregation, as well as for facilitating refolding and removal of damaged proteins [39]. Another gene, *OXSR1*, encodes an oxidative stress responsive protein, which plays an important role in response to oxidative stress [63]. Expression of this gene was also up-regulated in the liver of torpid bats. In addition, three KEGG pathways, proteasome (ko03050), ubiquitin mediated proteolysis (ko04120) and DNA replication (ko03030) were only represented by up-regulated genes in the torpid state, which indicates an increase in the processes of ubiquitin-mediated proteolysis and DNA replication during hibernation. An increase in the level of proteins conjugated to ubiquitin in tissues can be used as an indicator of protein damage induced by stress [64]. The accumulation of ubiquitin conjugates has been found in the gut and liver of torpid squirrels [65]. Protein ubiquitination is part of the ubiquitin-mediated proteolysis process, which involves the proteasome, so enhanced ubiquitin-mediated proteolysis may be a response to stress that leads to protein damage. Indeed, the over-expression of genes during torpor involved in DNA replication may function as a repair mechanism to reduce this risk. We infer increased expression of cell growth and death related genes regulating cell apoptosis (ko04210) and cell motility related genes regulating actin cytoskeleton (ko04810) to be associated with enchanced cell activies, which may be one of protective mechanisms during hibernation. Three signal transduction pathways, MAPK (ko04010), ErbB (ko04012) and Notch signaling pathways (ko04330) were significantly associated with up-regulated genes in the torpid state. These three pathways play an essential role in many aspects of cell regulation, such as cell proliferation, cell differentiation and cell death [66–68]. These enhanced cell regulation activities may help to eliminate damaged cells and replenish new cells to protect the liver from damage.

## Conclusion

In this study, the Illumina HiSeq 2000 platform was used to sequence the liver transcriptomes of active and torpid *R*. *ferrumequinum* in order to gain insights into changes of gene expression patterns in the liver during hibernation. Differentially expressed genes identified major involved in metabolic depression, shifts in the fuel utilization, immune function and response to stresses. Comparisons with other mammals, such as black bears, ground squirrels, and other bats, revealed some common transcriptional changes: genes involved in carbohydrate catabolism were down-regulated during torpor, while genes responsible for lipid β-oxidation were up-regulated. Similar transcriptional changes in mammalian hibernators would suggest that hibernation is physiologically similar in all mammals and it could be convergence. In addition, the depression of genes involved in glycolysis and induction of genes encoding heat shock proteins in the liver of hibernating *R*. *ferrumequinum*, consistent with studies on gene expression in the brain of hibernating bats. This may indicate that the presence of general changes in gene expression in the tissues of hibernating bat, however, further investigation on other tissues is needed. In this study, we also found immunity-associated genes were up regulated during torpor, which has been little reported before. The enhanced immune activity that we have inferred in the liver may help to protect bats from pathogen invasion. However, further studies are needed to understand whether immune gene up regulation is a general pattern and protective against pathogens. In general, this study provides comprehensive of transcriptional evidence of the physiological changes in the liver of *R*. *ferrumequinum* during torpor.

## Supporting Information

S1 Table Primers of 13 genes and *β-actin* gene (housekeeping gene) used for real time PCR. (DOCX)

S2 Table Down and up regulated genes in the livers of *R*. *ferrumequinum* during torpor. (XLSX)

S3 Table GO terms significantly annotated by differentially expressed genes. (XLSX)

S4 Table KEGG pathways significantly annotated by differentially expressed genes. (XLSX)
