# Supplementary material for: Differential Expression of Hepatic Genes of the Greater Horseshoe Bat (Rhinolophus ferrumequinum) between the Summer Active and Winter Torpid States
Source: PLoS One. 2015 Dec 23;10(12):e0145702. doi: 10.1371/journal.pone.0145702 (PMC4689453; doi:10.1371/journal.pone.0145702)
Supplement: S1 Table — (DOCX) [file pone.0145702.s001.docx]

| **Gene** | **Primer** | **Base sequence (5’- 3’)** | **Products (bp)** |
| --- | --- | --- | --- |
| *SDR42E1* | Sense | CTTCCTCTTGGTTATGGCAGTT | 319 |
|  | Anti-sense | AAGAAAGAGGGCACACAAGAAG |  |
| *CCND2* ^a^ | Sense | GAGATCCCCAAGAACTCT | 228 |
|  | Anti-sense | AGTCAGTCTCGTAGGGTG |  |
| *TXK* | Sense | CTGCCTGGTCACAGAGAAGAAC | 164 |
|  | Anti-sense | CATCGCTCTCGGACGAATAG |  |
| *ALAS2* | Sense | TGGGATTGGGGAGCGTGAT | 168 |
|  | Anti-sense | ATGGGAGGCAGTGAAGTGGTG |  |
| *NUP37* | Sense | AAACACCTTGAAAGTCGGAG | 166 |
|  | Anti-sense | GCCAGGATAACCAGTAGTAGC |  |
| *DYRK1A* ^b^ | Sense | TTATGACACACGCAAAGTGA | 192 |
|  | Anti-sense | GGCCTTATGGATTTGGA |  |
| *UFSP2* | Sense | CAGTGCTCAGACAGAAACGAAGT | 159 |
|  | Anti-sense | TGTTGAGCCAAAAGCCATAGC |  |
| *ZBED1* | Sense | TGACGTAGGTCCTGTTCTGGTT | 238 |
|  | Anti-sense | CTCATCTGCGATGGGCTGT |  |
| *TAT* | Sense | AACATCGGTGGGAAAAACT | 215 |
|  | Anti-sense | GTAACTTCTGGGTCTGTAGGC |  |
| *UCP2* | Sense | GCTGCTCATAGGTGACGAACAT | 196 |
|  | Anti-sense | ACCATCATTGCCTCCCCTGT |  |
| *FGA* | Sense | CATACCTGATTTAGACTCGTTCC | 170 |
|  | Anti-sense | CAGATGCTGAGCCCATAGAC |  |
| *ARG1* | Sense | TCCAAGCCCAAATCCATAGG | 153 |
|  | Anti-sense | CAGGTCCCCATAATCTTTTACATC |  |
| *CPB2* | Sense | AGCAGACTTCCAACGACGC | 271 |
|  | Anti-sense | AACCACAAGCAGAAAGCAGG |  |
| *β-actin* ^a^ | Sense | GACCTCTATGCCAACACAG | 190 |
|  | Anti-sense | CATCTGCTGGAAGGTGGAC |  |

^a^Primer pairs of *CCND2* and *β-actin* was obtained in Chen et al. 2008. ^b^Primers obtained in Chen et al. 2007.
